# Arachidonic Acid and Docosahexaenoic Acid Metabolites in the Airways of Adults With Cystic Fibrosis: Effect of Docosahexaenoic Acid Supplementation

**DOI:** 10.3389/fphar.2019.00938

**Published:** 2019-08-23

**Authors:** Elisabetta Teopompi, Patrizia Risé, Roberta Pisi, Carola Buccellati, Marina Aiello, Giovanna Pisi, Candida Tripodi, Valentina Fainardi, Enrico Clini, Alfredo Chetta, G. Enrico Rovati, Angelo Sala

**Affiliations:** ^1^Respiratory Disease Unit, Department of Medicine and Surgery, University Hospital, Parma, Parma, Italy; ^2^Department of Pharmacological and Biomolecular Sciences, University of Milan, Milano, Italy; ^3^Department of Pediatrics, CF Unit Children Hospital, University Hospital of Parma, Parma, Italy; ^4^Department of Medical and Surgical Sciences, University of Modena Reggio Emilia, Modena, Italy; ^5^Department of Medical and Surgical Sciences SMECHIMAI, University Hospital of Modena, Modena, Italy; ^6^IBIM, Consiglio Nazionale delle Ricerche, Palermo, Italy

**Keywords:** 15-lipoxygenase, sputum, inflammatory mediators, docosahexaenoic acid-DHA, arachidonic acid (AA or eicosatetraenoic acid)

## Abstract

Cystic fibrosis (CF) is an autosomal recessive disorder, caused by genetic mutations in CF transmembrane conductance regulator protein. Several reports have indicated the presence of specific fatty acid alterations in CF patients, most notably decreased levels of plasmatic and tissue docosahexaenoic acid (DHA), the precursor of specialized pro-resolving mediators. We hypothesized that DHA supplementation could restore the production of DHA-derived products and possibly contribute to a better control of the chronic pulmonary inflammation observed in CF subjects. Sputum samples from 15 CF and 10 chronic obstructive pulmonary disease (COPD) subjects were collected and analyzed by LC/MS/MS, and blood fatty acid were profiled by gas chromatography upon lipid extraction and transmethylation. Interestingly, CF subjects showed increased concentrations of leukotriene B_4_ (LTB_4_), prostaglandin E_2_ (PGE_2_), and 15-hydroxyeicosatetraenoic acid (15-HETE), when compared with COPD patients, whereas the concentrations of DHA metabolites did not differ between the two groups. After DHA supplementation, not only DHA/arachidonic acid (AA) ratio and highly unsaturated fatty acid index were significantly increased in the subjects completing the study (*p* < 0.05) but also a reduction in LTB_4_ and 15-HETE was observed, together with a tendency for a decrease in PGE_2,_ and an increase in 17-hydroxy-docosahexaenoic acid (17OH-DHA) levels. At the end of the washout period, LTB_4_, PGE_2_, 15-HETE, and 17OH-DHA showed a trend to return to baseline values. In addition, 15-HETE/17OH-DHA ratio in the same sample significantly decreased after DHA supplementation (*p* < 0.01) when compared with baseline. In conclusion, our results show here that in CF patients, an impairment in fatty acid metabolism, characterized by increased AA-derived metabolites and decreased DHA-derived metabolites, could be partially corrected by DHA supplementation.

## Introduction

Cystic fibrosis (CF) is an autosomal recessive disorder caused by genetic mutations in CF transmembrane conductance regulator (CFTR) protein (Cant et al., 2014). Defective CFTR causes impaired or absent transport of chloride through cell membranes, with an impaired mucociliary clearance and viscous mucus in the airways, which results in the inability of the airways to clear bacteria (Ciofu et al., 2013). Patients with CF experience declining pulmonary function related to chronic airway inflammation, which results from epithelial and immune cell secretion of proinflammatory mediators that promote neutrophil influx into the airways (Cantin et al., 2015). This inflammatory response results in a marked neutrophil infiltration with release of reactive oxygen species (ROS), pro-inflammatory lipid mediators (LMs), and proteases, including neutrophil elastase, with the final result of cleaving structural proteins, leading to bronchiectasis (McCarthy et al., 2014). Although new therapies may be able to target the underlying abnormality rather than its downstream effects (Prickett and Jain, 2015), modulating the airway inflammation in patients with CF may still provide relief and contribute to the management of the pathology (Jones and Helm, 2009; Ratjen, 2009). In fact, although the impact of CFTR modulators on lung function is exciting, they have not yet demonstrated an effect on inflammation; therefore, anti-inflammatories for the treatment of CF subjects are still needed.

Several reports have indicated the presence of specific fatty acid (FA) alterations in CF patients (Rivers and Hassam, 1975). When this observation was described more than 40 years ago, the primary abnormality identified was decreased linoleic acid (LA) levels in the plasma of CF patients. FA acid in the blood and tissues of CF patients, most notably decreased levels of DHA (Freedman et al., 1999), suggesting that FA alterations might play a role in the symptoms and progression of the CF disease (Freedman et al., 2004).

Several classes of DHA-derived LMs, arising from different lipoxygenases as well as aspirin-inactivated cyclooxygenase-2 (COX-2), such as resolvins (Serhan et al., 2002), protectins (Marcheselli et al., 2003; Serhan et al., 2011), and maresins (Serhan et al., 2009), and their aspirin-triggered epimers, have been identified as potentially important factors in the resolution phase of the inflammatory reaction (Serhan, 2014). These new compounds possess potent and specific activities in controlling the resolution of inflammation: the term resolvins, resolution phase interaction products, was introduced to indicate that the new structures are endogenous, local-acting mediators (autacoids) possessing potent anti-inflammatory, and immunoregulatory properties (Serhan et al., 2002). These include reduction of neutrophil infiltration and regulation of the cytokine–chemokine axis as well as the production of ROS. The protectin family, or neuro-protectin when of neural origin (Hong et al., 2003), was named after the potent anti-inflammatory and protective actions demonstrated in different animal models, such as stroke and Alzheimer’s disease (Lukiw et al., 2005).

Protectin D1 (PD1), and its precursor 17-hydroxy-docosahexaenoic acid (17OH-DHA), has been identified in exhaled breath condensates from healthy volunteers, while significantly lower concentrations were detected in exhaled breath condensates from asthmatic subjects (Levy et al., 2007), suggesting that endogenous PD1 may represent a counterregulatory signal in airway inflammation and suggesting new therapeutic strategies for the modulation of lung inflammation.

Recently, several studies have tested the efficacy of DHA supplementation in restoring the production of SPMs, mainly looking at the concentrations of SPMs in plasma, on the assumption that SPMs do actually circulate (Colas et al., 2014; Elajami et al., 2016), even if negative reports have also appeared in the literature (Fischer et al., 2014; Skarke et al., 2015). In the present study, we evaluated the concentrations of metabolites arising from both arachidonic acid (AA) and DHA at the relevant site of synthesis, that is, within the airways, using induced sputum from CF patients before and after supplementation with DHA, under the working hypothesis that DHA supplementation may boost the production of the pro-resolution metabolites, such as resolvins and protectins, while using the concentrations of eicosanoids as inflammatory markers of the ongoing inflammatory reaction within the airways of CF subjects, as well as for normalization of the samples. At the same time, changes in red blood cells (RBC) membranes FA profile were evaluated, to assess the efficacy of the 10 weeks supplementation in correcting the deficit of DHA observed in CF subjects. We also compared the concentrations of metabolites arising from both AA and DHA from CF patients to those from patients affected by chronic obstructive pulmonary disease (COPD), which is characterized by an acquired neutrophilic airway inflammation similar to that of CF subjects but in the presence of a normal plasmatic FA profile.

## Materials and Methods

### Subjects

CF subjects meeting inclusion criteria and providing informed consent were consecutively recruited over the period of 6 months (six males and nine females; age range, 20 to 40 years) at the Department of Medicine and Surgery, Respiratory Disease Unit and the Department of Pediatrics, Children Hospital of the University of Parma; 10 COPD patients (age range, 45 to 70 years; four males and six females), were recruited upon informed consent, at the Department of Oncology, Haematology, Respiratory Diseases and Ospedale Villa Pineta di Gaiato, Pavullo (MO). At baseline all subjects performed nutritional status evaluation, Shwachman-Kulczycki score evaluation, spirometry, sputum induction. The protocol was approved by the Ethical Committees of the Clinical institutions involved.

All patients with CF were diagnosed by evidence of CFTR dysfunction (elevated sweat test) and/or identification of two pathological CFTR mutations (INNO-LiPA CFTR19^®^). The inclusion criteria were: genotype ΔF508 homozigous, mild/moderate pulmonary disease (forced expiratory volume at the 1st second [FEV1] ≥40% predicted value), and pancreatic insufficiency. All patients were clinically stable and following standard CF therapy.

COPD subjects, GOLD stage 2 to 3 under treatment according to the GOLD document (Vogelmeier et al., 2017), were examined at the time of enrolment, whereas CF patients were examined both at enrolment; after 10 weeks of supplementation with Aladin^®^ 500 mg (Laborest, Italy), two capsules, three times a day; and after an additional 10 weeks of washout. Dosage and duration regimen adopted were those already routinely used by the CF hospital unit, and were based on average values present in the literature (Coste et al., 2007; Oliver and Watson, 2016). Nine CF patients (five female) completed the study.

All subjects recruited performed nutritional status evaluation, spirometry, and sputum induction (SI), whereas blood sampling for FA profiling was performed in CF subjects only. Sputum and blood samples were kept at –80°C until analysis.

The pulmonary functions forced vital capacity (FVC) and FEV_1_ was measured with a spirometer and a body pletismograph (B3Box Biomedin, Padua, Italy), and oxygen saturation (SaO_2_) was measured by pulse oxymetry (Nellcor N-395).

### Sputum Collection

Induced sputum collection was performed in accordance with the European Respiratory Society task force (Efthimiadis et al., 2002; Paggiaro et al., 2002). FEV_1_ and FVC were measured at baseline and after inhalation of salbutamol (200 µg by metered dose inhalers). After that, subjects were asked to rinse their mouth before inhalation of sterile hypertonic saline (NaCl, 3%, prepared by the hospital chemist) nebulized with an ultrasonic device (Heyer Orion 1, BAD EMS; mean volume output: 2.40 ml/min) for four cycles of 5 min each. After each cycle and when needed, they were asked to rinse their mouth and cough into a plastic container. Three flow volume curves were performed before and after each inhalation, and the best FEV_1_ was recorded. Induction of sputum was stopped if FEV_1_ value fell by at least 15% from baseline or if troublesome symptoms occurred. The collected sputum samples were processed as previously described (Kodric et al., 2007). The volume of the sputum sample was measured, and an equal volume of dithiotreitol 0.1% was added and incubated at 37°C for 30 min. Ten microliters of the homogenized sample was used to determine the total and differential cell count, expressing the result as number of cells/ml and % of total cells, respectively. The remaining sputum was centrifuged at 400*g* for a 5-min period. The supernatant was aspirated and stored for LC/MS/MS analysis.

### Mass Spectrometry of AA and DHA Metabolites

After thawing, sputum supernatant samples (0.2-1 ml) were added with stable isotope labeled internal standards ([d_4_]LTB_4_, [d_4_]PGE_2_, [d_8_]5-HETE, and [d_5_] lipoxin A_4_ (LXA_4_) 2.5 ng each), centrifuged to remove particulate, acidified with acetic acid (final concentration 0.01%) and extracted using preconditioned polymeric solid phase extraction cartridges (Strata-X, 33 µm Polymeric Reversed Phase; Phenomenex, Torrance, CA). After washing with ultrapure water, DHA- and AA-derived metabolites were eluted using methanol/water, 90/10, v/v (0.5 ml), and the eluate taken to dryness using a rotary vacuum evaporator (SpeedVac; Thermo Scientific, Waltham, MA). Upon reconstitution in 40 µL HPLC solvent A (8.3 mM acetic acid buffer to pH 5.7 with ammonium hydroxide) plus 20 µl of HPLC solvent B (acetonitrile/methanol, 65:35, v/v), an aliquot of each sample (20 µl) was injected onto a C18 HPLC column (Ascentis 150 × 2 mm, 3 µm; Supelco, Bellefonte, PA) and eluted at the rate of 400 µl/min with a linear gradient from 45% solvent B, which was increased to 75% in 12 min, to 98% in 2 min, then held for 11 min before re-equilibration at 45% B for 10 min. The HPLC effluent was directly infused into an triple quadrupole mass spectrometer (6460, Agilent) equipped with electrospray ion source for mass spectrometric analysis in the negative ion mode using multiple reaction monitoring (MRM) for the specific *m/z* transitions: 343-281 for 17OH-DHA (the precursor of both resolvins and protectin), 359-206 for PD1, 375-141 for resolvin D2 (RvD2), 335-195 for LTB_4_, 319-219 for 15-HETE, 351-271 for PGE_2_, 327-116 for [d_8_]5-HETE, 339-197 for [d_4_]LTB_4_, 359-275 for [d_4_]PGE_2_, and 356-222 for [d_5_]LXA_4,_ that was used as IS for RvD2 (Pioselli et al., 2010). Quantitation was performed using isotope dilution of the internal standards, and data were analyzed using MassHunter software. Standard curves were obtained using synthetic PD1 (a gift from Dr. Thierry Durand, CNRS, Montpellier, France), LTB_4_, PGE_2_, RvD2, 15-HETE, and 17OH-DHA (Cayman Chem, Ann Arbor, MI). The peak–area ratios of every compound to the relevant deuterated internal standard was calculated and plotted against the amount of the synthetic standards. Calibration lines were calculated by the least squares linear regression method and the correlation coefficient r^2^ was always better than 0.99. To calculate the concentration of any given analyte, the peak–area ratio to the relevant internal standard was calculated and read off the corresponding calibration line. Detection limit varied between 1 and 25 pg injected (3 to 75 pg in the sample), depending on the analyte.

Optimization of declustering potential, collision energy and CXP, was carried out for each metabolite directly injecting 1 to 5 ng of synthetic standard using the same eluent used for the analysis.

### FA Analysis

Blood samples were collected in 10% sodium heparin and centrifuged at 200*g* for 18 min.

The lower fraction was additionally centrifuged at 800*g* for 18 min, and the pellet washed twice with phosphate buffer containing 0.1 M NaH_2_PO_4_ (5:1 v/v). Cells were lysed with water, followed by washing (twice) to obtain the RBC membranes for the lipid extraction. Total lipids (TL) were extracted according to Folch. Briefly, 5-ml chloroform-methanol 2:1 will be added and homogenized with a Politron, followed by 1 ml phosphate buffered saline. After 2 h at -20°C, the organic phase of samples will be evaporated under a stream of nitrogen, and a volume of 2:1 chloroform:methanol solution containing butylatedhydroxytoluene (5 mg/ml) as antioxidant was added. The lipid concentration of the extracts was determined by microgravimetry.

FA methyl esters prepared by acid transmethylation and analyzed by gas–liquid chromatography (GC 2010 Shimadzu), using a capillary column of 15 m, 0.1 I.D., 0.1 µm film (DB-FFAP, Agilent); temperature was programmed from 130°C to 220°C, and peaks were identified using pure reference compounds. Data were expressed as percentage of total amounts of FAs.

### Data Analysis

Experimental values were expressed as the mean and standard error of the mean (SEM). Statistical analysis was performed using *t*-test for paired or unpaired data, or one-way ANOVA followed by Dunnett’s *t* test as appropriate.

## Results

LC/MS/MS analysis of sputum samples detected significant amounts of several LMs, such as LTB_4_, PGE_2_, and 15-HETE, as well as 17OH-DHA, which represents the precursor of protectin and resolvins; RvD2 could be detected at a s/n ratio above 10 only in a sample from the COPD basal group (1.7 ng/ml), but in none of the CF samples obtained before supplementation with DHA. PD1 was also only detected in 6 of 10 COPD subjects, with an average concentration, in these positive subjects, of 400 ± 220 pg/ml.

**Figure 1:**
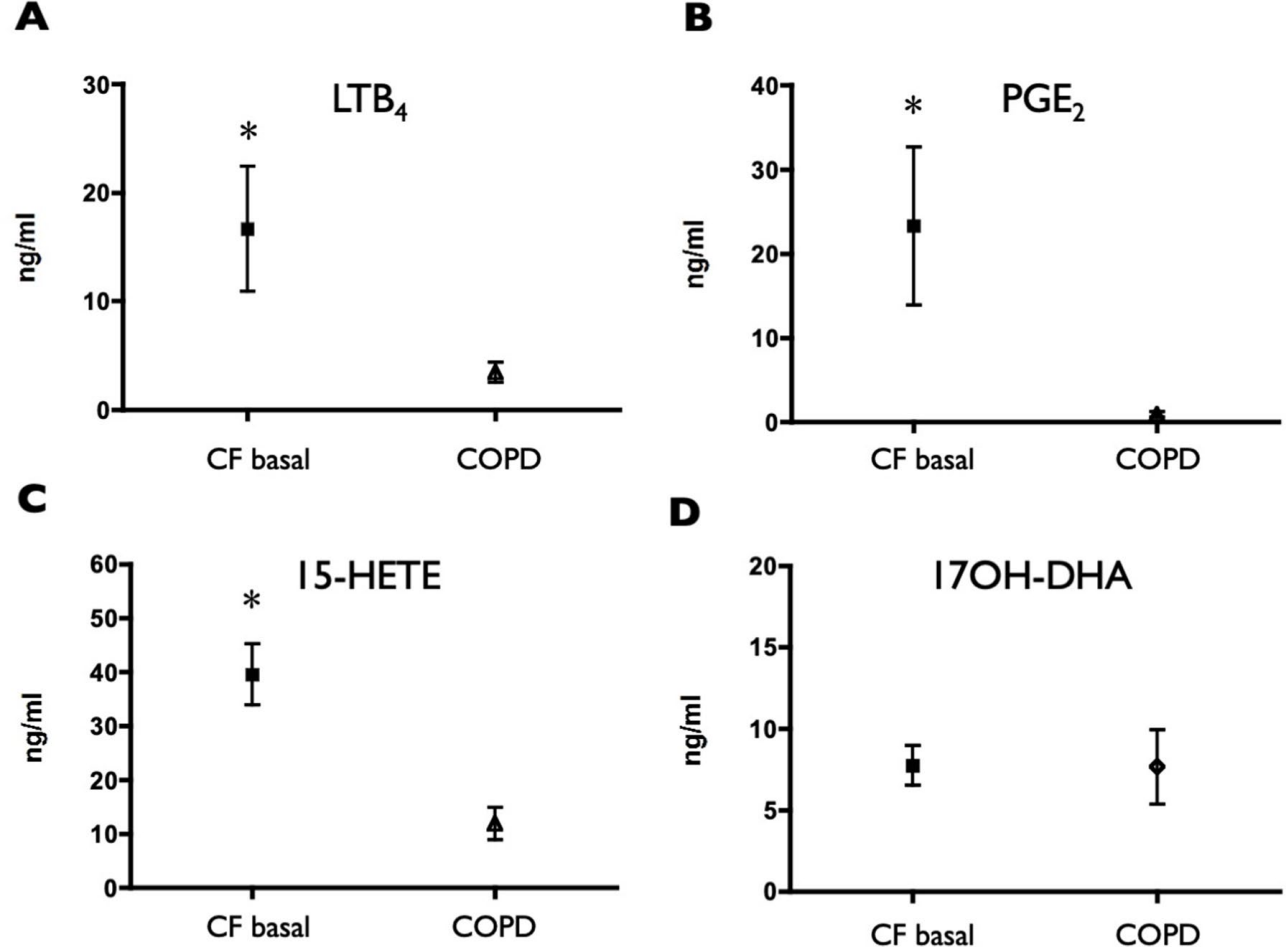
Induced sputum supernatant concentrations of LTB_4_ **(A)**, PGE_2_ **(B)**, 15-HETE **(C)**, and 17OH-DHA **(D)** in CF subjects and in COPD subjects. Lipid mediators were quantitated by LC/MS/MS as described in *Materials and Methods*. Data were analyzed with *t* test for unpaired data and are expressed as mean ± SEM (n = 15 for CF and n = 10 for COPD); *p < 0.05 vs COPD.

Comparing the values observed in CF subjects with those of COPD subjects, showed markedly higher values of LTB_4_, PGE_2_, and 15-HETE in CF subjects (**Figure 1A–C**). Surprisingly, the concentrations of the precursor of resolvins/protectins 17OH-DHA resulted remarkably similar in the two groups, therefore unmasking a rather large unbalance toward AA-derived, mainly pro-inflammatory mediators over the potentially pro-resolving DHA-derived metabolites in CF subjects (**Figure 1D**).

Analysis of FA composition before and after 10 weeks of DHA supplementation was performed in eight of the nine CF subjects that completed the study (one blood sample could not be collected) and showed a significant increase in the DHA/AA ratio, and in the n-3 highly unsaturated FA (HUFA) index, that is the percentage of n-3 FAs over the total amount of HUFA present in RBCs phospholipids. The FA composition of RBC membranes was used as it reflects fat intakes but since RBCs have a rather long lifespan, their FA profile is considered a better long-term marker of FA intake within a middle term time period (from 3 weeks to 3 months) than platelet or plasma lipids (Stanford et al., 1991; Theret et al., 1993).

**Figure 2:**
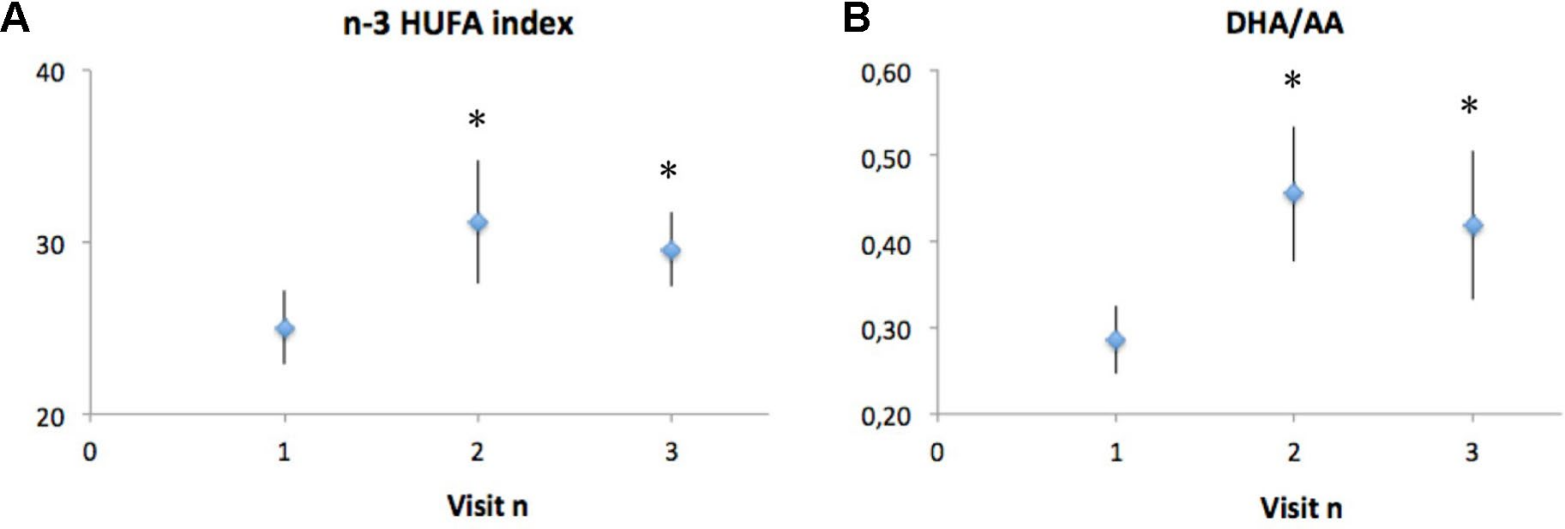
Analysis of fatty acid composition in CF subjects before (visit 1) and after (visit 2) 10 weeks of DHA supplementation, and 10 weeks after the end of DHA supplementation (visit 3). Polyunsaturated fatty acid composition is reported as the n-3 HUFA index **(A)**, which is the percentage of n-3 fatty acids over the total amount of HUFA present in red blood cells, as well as DHA/AA ratio **(B)**, which is the percentage of n-3 fatty acids over the total amount of HUFA present in red blood cells. Data are expressed as mean ± SEM (n = 8). Statistical analysis was carried out by ANOVA repeated measure; *p < 0.05.

The observed changes were still noticeable at the end of the 10 weeks washout period, reflecting a lasting effect on membrane phospholipid FA composition, even if a trend toward basal values was present (**Figure 2**).

**Figure 3:**
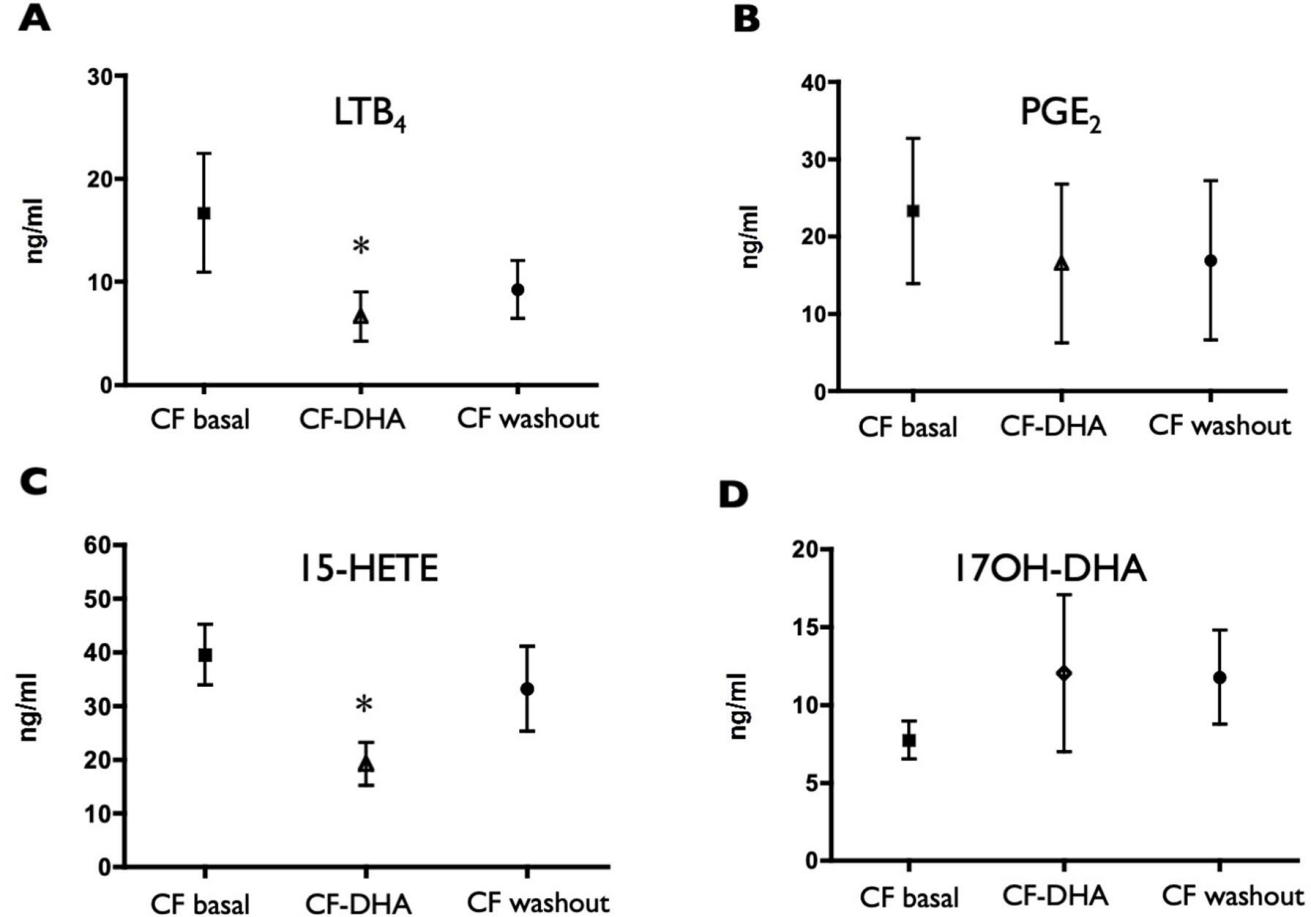
Induced sputum supernatant concentrations of LTB_4_ **(A)**, PGE_2_ **(B)**, 15-HETE **(C)**, and 17OH-DHA **(D)** in CF subjects before (CF basal) and after (CF-DHA) 10 weeks of DHA supplementation, and 10 weeks after the end of DHA supplementation (CF washout). Lipid mediators were quantitated by LC/MS/MS as described in *Materials and Methods*. Data were analyzed with ANOVA followed by Dunnett’s test, and are expressed as mean ± SEM (n = 9-15); *p< 0.05 vs CF basal.

**Figure 4:**
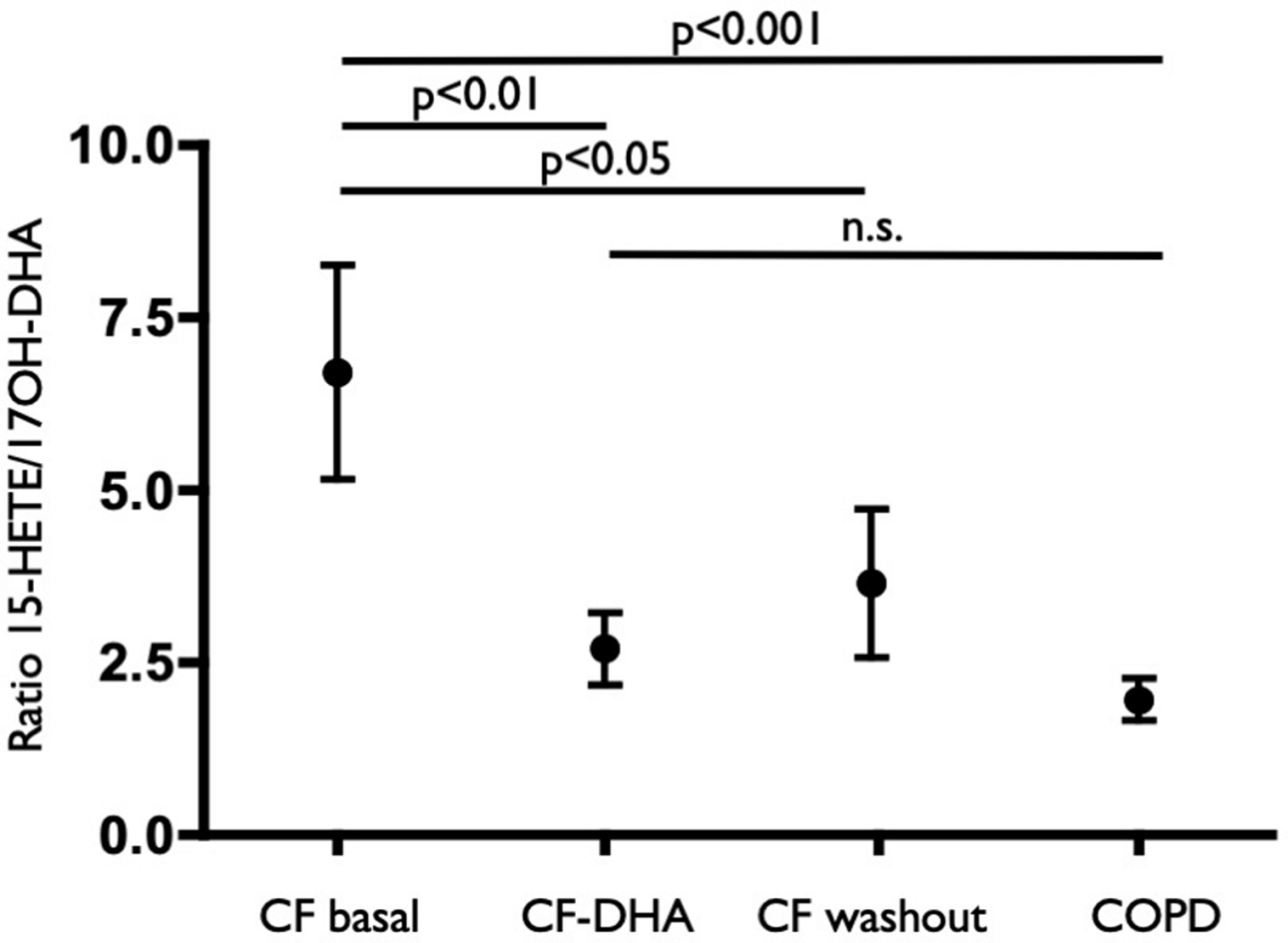
Ratio values of the concentration of 15-HETE and 17OH-DHA in each sample from COPD subjects (COPD), CF subjects before (CF basal) and after (CF-DHA) 10 weeks of DHA supplementation and 10 weeks after the end of DHA supplementation (CF washout). Lipid mediators were quantitated by LC/MS/MS as described in Materials and Methods. Data were analyzed with ANOVA followed by Dunnett’s test and were expressed as mean ± SEM (n = 9-15 for CF and n = 10 for COPD).

The sputum concentrations of LTB_4_ and 15-HETE decreased at the end of the DHA supplementation (**Figures 3A, C**) and remained lower, on average, at the end of the washout period, even if with a trend to recover pre-supplementation values. A similar trend (although less pronounced and not statistically significant) was observed for PGE_2_ (**Figure 3B**), whereas 17OH-DHA showed increased concentrations at the end of the 10 weeks of supplementation with (**Figure 3D**), generating a significant correction of the unbalance between AA- and DHA-derived LMs observed under basal conditions. This correction was particularly evident when focusing on 15-HETE and 17OH-DHA, metabolites arising from the same enzymatic activities, namely, 15-LOX or aspirin-inactivated COX-2, on AA and DHA, respectively. Indeed, 10 weeks of supplementation with DHA significantly decreased the ratio between the concentrations of 15-HETE and 17OH-DHA observed in the same sputum sample to values that were not different from those observed in COPD subjects (**Figure 4**). Interestingly, RvD2 could be detected at the concentrations of 570 and 283 pg/ml in two FC samples obtained after DHA supplementation. PD1 could not be quantitated in any sample from FC subjects, either before or after DHA supplementation.

Differential cell count showed a non-statistically significant decrease in neutrophils upon DHA supplementation, which rapidly reversed upon washout (before supplementation 80.2 ± 8.1%; after supplementation 61.5 ± 31.5%; after washout 78.7 ± 12.1%).

No statistically significant differences in pulmonary functions were observed after 10 weeks of DHA supplementation (FEV_1_ before supplementation, 69.8 ± 19.9% of predicted; after supplementation, 75.6 ± 19.4%; after washout, 76 ± 21%).

## Discussion

The link between the genetic defects in CFTR and inflammation/chronic bacterial infection is still unclear, but mutations in CFTR, together with the resulting limitation of water movement across the epithelium causing an impaired mucociliary clearance, may also affect the innate immune response associated with the epithelial cells, causing enhanced and ineffective inflammatory response that fails in controlling bacterial infections (Cohen and Prince, 2012).

DHA-derived LMs, such as resolvins, maresins, and protectin, collectively defined as part of the SPMs genus, could play a critical role in the resolution phase of the inflammatory reaction, enhancing clearance of microorganisms, and promoting tissue repair (Chiang and Serhan, 2017). It has been demonstrated that SPMs can modulate viral and bacterial infections, increasing phagocytosis and the ability to kill bacteria (Russell and Schwarze, 2014). Interestingly, PD1 and its immediate precursor 17-hydroxy-docosahexaenoic acid (17OH-DHA) have been identified in exhaled breath condensates from normal subjects, whereas lower concentrations were observed in subjects with asthma exacerbations (Levy et al., 2007), suggesting a potential role for PD1 as a potential modulator of airway inflammation and pointing to a novel therapeutic approach to modulate inflammation in the lung. On the other hand, AA metabolites have long been known as an hallmark of the inflammatory response, even if their specific contribution to the chronic inflammation observed in CF patients has not been clearly established (Reid et al., 2007).

Analysis of AA and DHA-derived LMs in sputum samples from CF subjects was performed under the hypothesis that the supplementation with DHA may boost the formation of DHA-derived anti-inflammatory/pro-resolution LMs when compared with AA-derived pro-inflammatory mediators, the latter used as marker of the ongoing chronic inflammation within the airways of CF subjects. Such analysis, in turn, also allows and inter-samples normalization that may result critical in samples with high variability, such as induced sputum supernatants.

**Figure 5:**
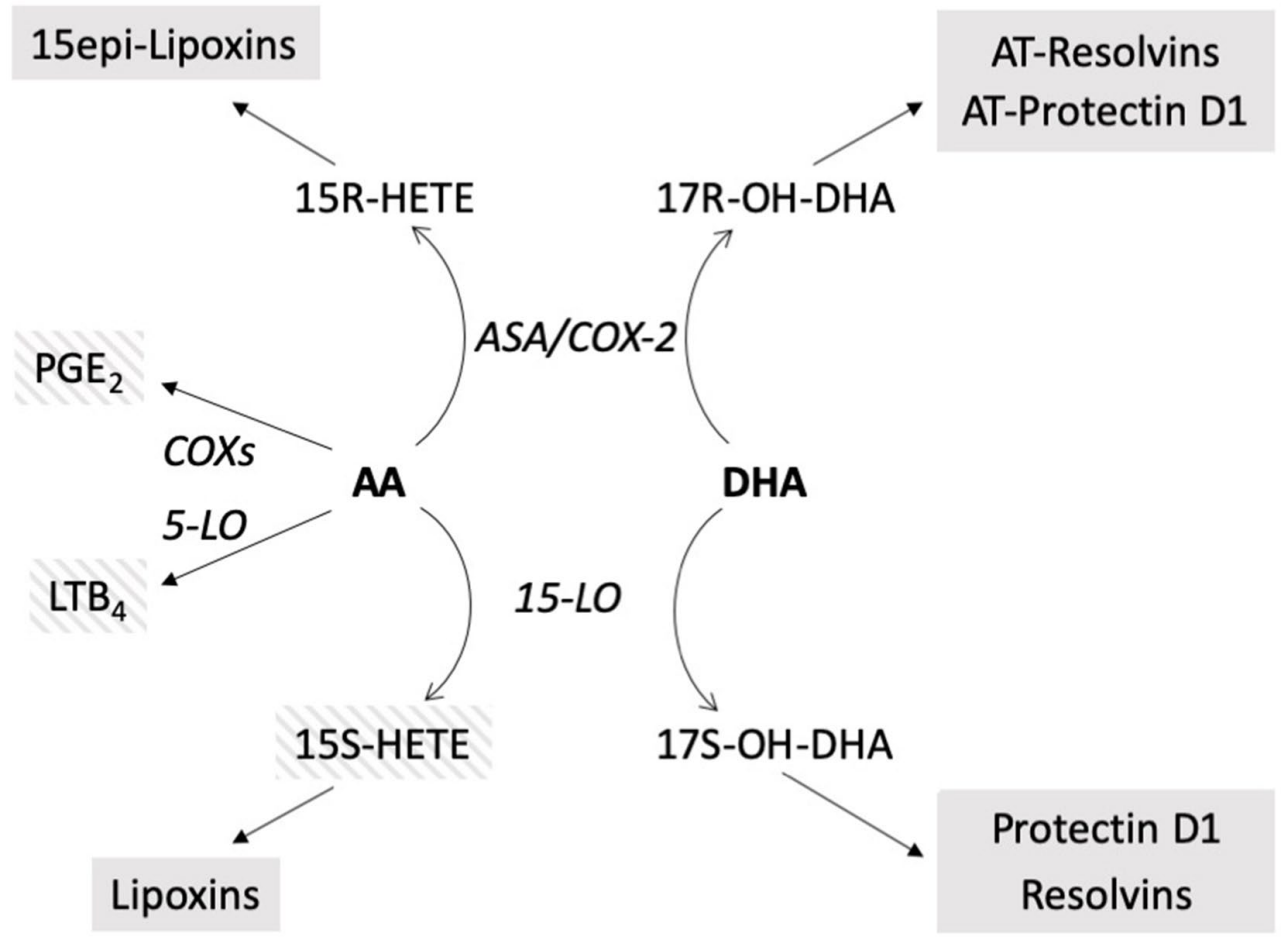
Schematic of the biosynthetic pathways of the compounds evaluated. In bold: precursors. In italics: enzymatic activities. In shaded boxes: biologically active compounds; striped: pro-inflammatory, solid: anti-inflammatory, pro-resolution. AT resolvins, AT protectin: aspirin-triggered resolvins, protectin (Weylandt et al., 2012), *ASA/COX-2*, aspirin acetylated COX-2.

A small group of COPD subjects was used as a comparator based on the observation that airway inflammation in both CF and COPD subjects is showing similar patterns, such as repeated infections and mostly neutrophilic inflammatory infiltrates, but with the COPD subjects lacking the altered profile of long-chain polyunsaturated FAs that is present in CF subjects. Although there are significant limitation associated with the comparison with COPD subjects (i.e., there is no possibility to match age between the two groups), the results obtained in CF subjects when compared with COPD patients showed a marked unbalance between pro-inflammatory mediators derived from AA and 17OH-DHA, that represents the precursor of protectins and resolvins, with both higher concentrations of pro-inflammatory LTB_4_ and PGE_2_, and lower concentrations of 17OH-DHA in CF subjects. Potential limitation in sensitivity inherent with the instrumentation used prevented the systematic assessment of biologically active protectins and resolvins in most samples, but it is interesting to note that they could only be detected in a limited number of samples in COPD subjects only. No chiral analysis was carried out for the mono-hydroxy derivative 17OH-DHA, but it must be noted that both the isomers, 17S and 17R, are precursors of compounds with significant biological activities, that is, resolvins/protectins and aspirin-triggered resolvins/protectins, respectively (**Figure 5**) (Serhan et al., 2004; Weylandt et al., 2012).

Correlations between altered FA levels and genotype, pancreatic status, and respiratory deficiency have been previously reported in CF subjects (Strandvik et al., 2001; Coste et al., 2008; Maqbool et al., 2008; Rise et al., 2010), and evidence has also been provided that a prolonged n-3 FA supplementation may have some positive effects on CF (Oliver and Watson, 2016). In our study, DHA supplementation resulted in a decrease of pro-inflammatory mediators, such as LTB_4_, suggesting that the lower concentrations of DHA observed in CF subjects (Rise et al., 2010) could indeed cause a lack of anti-inflammatory/pro-resolving LMs. 15-HETE also showed a significant decrease, but its role is more complex in that while possessing pro-inflammatory activities on the airways (Johnson et al., 1985; Li et al., 2013), it may also serve as precursor of anti-inflammatory lipoxins (Serhan, 1989) (see **Figure 5**), and therefore the net change associated with its decrease could be nil. Nevertheless, the concentrations of 15-HETE can be used, as we did, as a normalizing factor, as they could be the result of the same enzymatic activities generating 17OH-DHA, namely 15-LO (for the S isomers) or aspirin-inactivated COX-2 (for the R isomers). In a biological sample characterized by a significant variability, such as induced sputum, absolute changes could be the result of different sampling rather than actual differences between patients conditions, whereas the assessment of relative changes, such as in the case of the ratio 15-HETE/17OH-DHA, is substantially immune from differences associated to changes in sampling.

The association between DHA supplementation, increased concentrations of the precursor of resolvins/protectins, and lower concentrations of pro-inflammatory LMs, such as LTB_4_, together with a tendency to a decrease in neutrophil infiltration also observed upon supplementation, provides evidence in support of a potential anti-inflammatory activity of n-3 PUFA supplementation in CF subjects. Interestingly, despite potential limitation in sensitivity of the mass spectrometer used that prevented the assessment of SPMs in most samples, it is worth noting that RvD2 was only detected in two samples obtained after supplementation with DHA, suggesting that indeed the increase in the precursor 17OH-DHA may be associated with an enhanced formation of SPMs.

The lack of clinical parameter amelioration observed is not surprising and could be due to the limited time of supplementation; the efficacy of n-3 FA supplementation has previously been reported as controversial due to different doses and preparations used, duration of supplementation, and differences in populations studied. Hanssens et al. found a reduced number of exacerbations and a decrease of the duration of antibiotic therapy after 9 months of n-3 supplementation in the absence of changes in lung function (Hanssens et al., 2016); on the contrary, De Vizia et al. reported an improvement of FEV_1_ not confirmed by others (De Vizia et al., 2003; Van Biervliet et al., 2008; Alicandro et al., 2013). Altogether, the results reported in these studies suggest that clinical parameters could be affected only after longer period of n-3 supplementation than those used in this study (i.e., 9 months to 1 year). In fact, anti-inflammatory treatments that were shown to benefit CF subjects, such as high-dose ibuprofen, were assessed on long-term function decline rather than immediate improvement of pulmonary parameters (Lands et al., 2007; Cantin et al., 2015).

In conclusion, our results provide preliminary evidence that the alterations in the FA profile observed in CF patients may result in a decrease in DHA-derived metabolites such as the SPMs, as suggested by comparison to COPD patients, who are affected by an acquired neutrophilic airway inflammation in the absence of plasmatic FAs alterations. This impairment was partially and reversibly corrected by DHA supplementation, which caused a simultaneous tendency toward a decrease of AA-derived metabolites and an increase in precursor to protectin and resolvin SPMs, together with a trend toward a decrease in neutrophil infiltration. Even with all the limitations of a small, pilot study, the results of this study support additional studies on the use of n-3 PUFA supplementation in CF subjects and suggest a potential usefulness of therapeutic approaches based on the local treatment with n-3 PUFA-derived endogenous anti-inflammatory molecules.

## Data Availability

The datasets generated for this study are available on request to the corresponding author.

## Ethics Statement

The protocol was approved by the Ethical Committees of the Clinical institutions involved: Children Hospital of the University of Parma; Ospedale Villa Pineta di Gaiato, Pavullo (MO). Informed consent was obtained from all participating subjects.

## Author Contributions

AS, MA, EC, GP, and AC conceived and designed the experiments. ET, RP, CB, CT, VF, and PR performed the experiments. AS, ET, GER, and PR analyzed the data. AS participated in the paper drafting. MA, GP, EC, AC, GER, PR, and AS participated in paper revision and intellectual contributions.

## Funding

This work is supported by the Fondazione per la ricerca sulla Fibrosi Cistica grant N. 17/11.

## Conflict of Interest Statement

The authors declare that the research was conducted in the absence of any commercial or financial relationships that could be construed as a potential conflict of interest.
